# Investigation of the Antibacterial Properties of Silver-Doped Amorphous Carbon Coatings Produced by Low Pressure Magnetron Assisted Acetylene Discharges

**DOI:** 10.3390/ijms23010563

**Published:** 2022-01-05

**Authors:** Valentin Job, Julie Laloy, Vincent Maloteau, Emile Haye, Stéphane Lucas, Sébastien Penninckx

**Affiliations:** 1 LARN Laboratory (LARN-NARILIS), University of Namur, 61 Rue de Bruxelles, B-5000 Namur, Belgium; valentin.job@unamur.be (V.J.); emile.haye@unamur.be (E.H.); sebastien.penninckx@unamur.be (S.P.); 2 Laboratory (NNC-NARILIS), Department of Pharmacy, University of Namur, 61 Rue de Bruxelles, B-5000 Namur, Belgium; julie.laloy@unamur.be (J.L.); vincent.maloteau@unamur.be (V.M.); 3 Innovative Coating Solutions (ICS), University of Namur, 11 Place Saint Pierre, B-5380 Forville, Belgium; 4 Medical Physics Department, Institut Jules Bordet, Université Libre de Bruxelles, 1 Rue Héger-Bordet, B-1000 Brussels, Belgium

**Keywords:** antibacterial coating, a-C:H, Ag, nanomaterials, diffusion, nosocomial infections, *S. aureus*, *E. coli*

## Abstract

Hospital-acquired infections are responsible for a significant part of morbidity and mortality. Among the possible modes of transmission, this study focuses on environmental surfaces by developing innovative antibacterial coatings that can be applied on interior fittings in hospitals. This work aims to optimize a coating made of an amorphous carbon matrix doped with silver (a-C:H:Ag) produced by a hybrid PVD/PECVD process and to evaluate its antibacterial activity. We present a coating characterization (chemical composition and morphology) as well as its stability in an ageing process and after multiple exposures to bacteria. The antibacterial activity of the coatings is demonstrated against *Escherichia coli* (Gram-negative) and *Staphylococcus aureus* (Gram-positive) bacteria through several bioassays. Moreover, the data suggest a crucial role of silver diffusion towards the surface and nanoparticle formation to explain the very promising anti-bacterial activities reported in this work.

## 1. Introduction

Healthcare-acquired infections, called nosocomial, are a major public health concern due to the associated morbidity and mortality [1]. The prevalence rate in industrialized countries varies from 3.5 to 12%, while rates of 18% have been reported in underdeveloped countries [2]. In Europe, this corresponds to more than 4 million affected people and approximately 40,000 deaths each year, making them the sixth leading cause of death [3]. In addition to the social dimension, these infections are a financial burden for our society, with direct financial losses estimated at €7 billion each year [4].

The pathogens responsible for these nosocomial infections are mainly bacteria, with Gram-positive *Staphylococcus aureus* being the most predominant [5]. Although bacteria can be transmitted by healthcare workers due to poor hand hygiene, contaminated surfaces on hospital fittings and equipment in the patients’ environment (beds, surgery instruments, door handles, etc) remains the major channel of transmission, estimated to be responsible of one third of all the infections [5,6,7,8,9]. Bacteria are thus transmitted from healthcare workers to patients in an indirect contact mode using these fomites.

To deal with this issue, the development of antimicrobial coatings is growing in order to impart the desired antibacterial surfaces properties without affecting the bulk properties of any objects. They can be classified in three categories based on their mechanism of action: (i) contact-killing coatings based on the interaction of bacterial membranes with macromolecules (such as chitosan) capable of disrupting lipid organization [10]; (ii) anti-adhesion coatings which prevent the formation of biofilms [11]; (iii) antibacterial agent release coatings which exert their action by leaching out compounds capable of killing bacteria [12].

Compared to the first two categories, the advantage of the release approach is to provide the anti-infectious agent locally, while avoiding any adverse toxic impact for human beings. Developed within the framework of nanomedicine [13,14,15,16,17], materials based on silver (Ag) ion release have been among the most studied candidates for antibacterial purposes because Ag hinders the formation of biofilm and kills bacteria whatever the state it is in (ions, nanoparticles, colloids [18]). In fact, it has been reported that silver nanoparticles (AgNPs) can infiltrate the cytoplasmic membrane, impacting both its permeability and fluidity [19]. Ag^+^ ions can inhibit a variety of antioxidant enzymes through their high affinity for thiols and amine groups constituting their active sites. This decreases the antioxidant defense systems by triggering an oxidative stress due to concomitant catalytic generation of reactive oxygen species [20]. Studies have also reported that these enzymatic inhibitions are also associated with a disruption in ATP production and a slowdown in DNA replication [21,22].

Nowadays, hydrogenated amorphous carbon films (a-C:H) are widely used as protective and decorative coatings. These films exhibit many useful properties such as very low friction coefficients, high wear resistance, chemical inertness and black color when thick enough. They can be applied on metals, polymers or ceramics of any shape and are extensively used in biomedical applications [23]. Surprisingly, they are very rarely involved in hospital furniture or hardware (e.g., door handles) even though they could be doped with microbicidal species such as Ag, which has already been incorporated in bactericidal paint [24] or healthcare personal clothes [25].

The purpose of this work is to study Ag release from a-C:H films (a-C:H:Ag) and evaluate its toxicity against Gram-positive and Gram-negative bacteria. Such films could possibly be applied on surfaces installed in the vicinity of hospitalized patients. Within this framework, a mature deposition technique compatible with an industrial application was chosen for evaluation of the antibacterial activity, in particular when successive washing and bacterial exposure is performed.

## 2. Results

### 2.1. a-C:H:Ag Deposition and Physical Characterisation

#### 2.1.1. Coating Production and Film Properties

Hydrogenated amorphous carbon coatings doped with silver were synthesized via a low pressure magnetron assisted discharge of C_2_H_2_ as reported in [26]. Typically, the films are amorphous and they are smooth (<10 nm for 500 nm thickness). The coated samples were deposited on stainless steel coupons, with different silver concentrations in the bulk of the layer [Ag(5.5%)], [Ag(6%)] and [Ag(22%)], the silver concentration being tuned by changing the reactive gas amount. These also contained a fixed amount of hydrogen (30%) and oxygen (1–2%).

**Figure 1:**
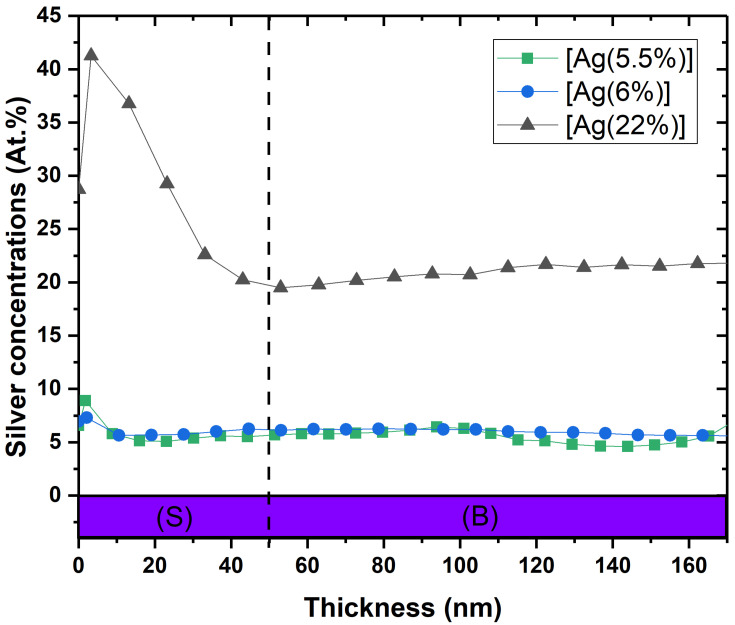
XPS depth profile of [Ag(5.5%)], [Ag(6%)] and [Ag(22%)] showing the atomic percentages (at.%) of silver in the a-C:H matrix containing Ag. (S) = surface ranging from 0 to 50 nm; (B) = bulk.

Figure 1 illustrates the depth chemical composition obtained by XPS profiling for 3 samples: [Ag(5.5%)], [Ag(6%)] and [Ag(22%)]. As it can be seen, the Ag atomic proportion (at.%) varies according to the depth of the layer in 2 steps. In the first segment (S), ranging from 0 to 50 nm, the increase in silver is greater for [Ag(22%)] than for the samples with lower silver concentrations. A maximal silver concentration of 42% and 9% was reported for [Ag(22%)] and [Ag(5.5%)], respectively. The second segment (B) is a portion with an almost constant concentration in bulk between 50 nm and 160 nm of depth profile.

#### 2.1.2. SEM Analyses and Silver Concentration Evolution at the Surface of Coated Samples

**Figure 2:**
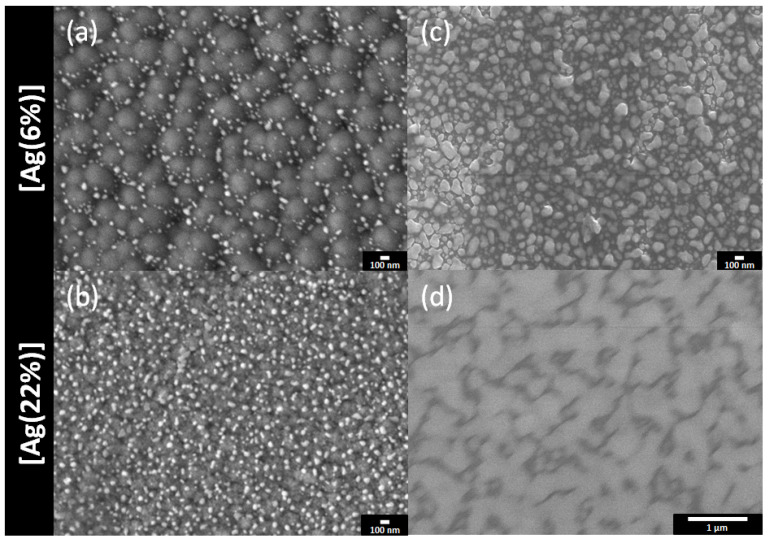
SEM surfaces images of a-C:H:Ag coatings: [Ag(6%)] on the top and [Ag(22%)] on the bottom. Top views of the coated samples directly after (**a**,**b**) and 1 year after (**c**,**d**) the deposition.

The top surfaces and the film cross sections of the samples were examined by using SEM. The results for [Ag(6%)] and [Ag(22%)] are shown in Figure 2, just after deposition (Figure 2a,b) and after a 1 year storage in a drawer (Figure 2c,d). For the deposited films, white clusters or nanoparticles are observed on the top surface. They reveal a grainy surface with black carbon polygons of approximately 328 nm, decorated with white bright silver nanoparticles in the periphery. The latter have an unregular morphology, indicating a surface segregation of silver in the grain boundary zones. Table 1 tabulates the AgNP dimensions. The mean Feret diameter is 59 nm, whereas the carbon surface covered by the silver clusters is 4.8%, with a particle density of 49 Part./µm^2^ for the [Ag(6%)]. For the [Ag(22%)] sample, the silver clusters are more uniformly distributed over the whole area and the surface does not exhibit carbon polygons. As indicated in Table 1, the density (188Part./µm^2^) and the covered surface (14.4%) are greater for the [Ag(22%)] than for the [Ag(6%)] sample, while the Feret diameter is similar.

The same samples were re-examined after 1 year. The SEM images (Figure 2c,d) show that the surfaces are almost totally covered in silver, either as large particles or as a film. The black polygons have disappeared (Figure 2c) and silver nanoparticles with a mean Feret diameter of 115 nm are observed. Figure 2d reveals the formation of an Ag film due to an agglomerate of silver nanoparticles. In both cases, the Ag content has increased and is approximately at 36% for [Ag(6%)] and 80% for [Ag(22%)], indicating a silver surface enrichment called surface segregation.

#### 2.1.3. Chemical Depth Composition and Ageing Behaviour

**Figure 3:**
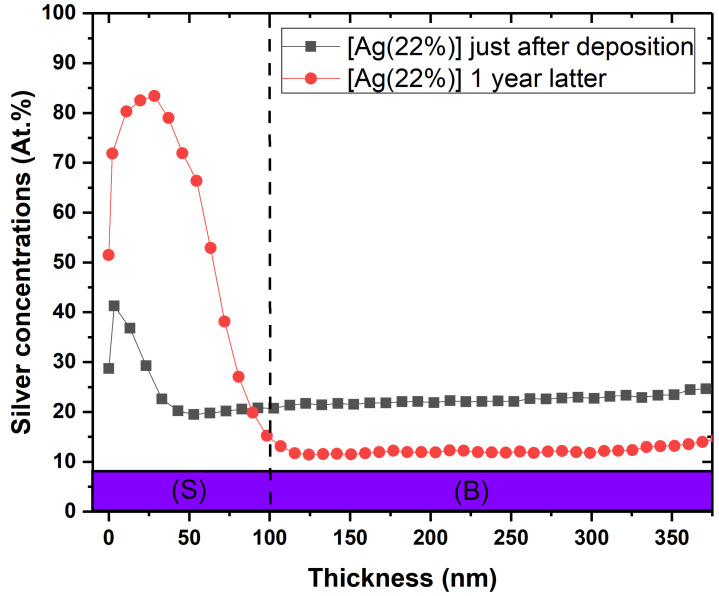
Ageing effect on silver depth concentration of the antimicrobial layer for [Ag22%)]. These depth chemical compositions were obtained by XPS analyses just after deposition (⯀) and one year later (●). (S) = surface ranging from 0 to 50 nm; (B) = bulk.

The SEM data were correlated with the silver depth distribution in the layers. Figure 3 shows the surface Ag concentration versus the depth for the [Ag(22%)] coated sample, just after deposition (⯀ + grey line) and one year later (● + red line). The comparison between the two analyses shows a strong diffusion of silver towards the surface (S), reducing the at.% in the bulk (B) to the benefit of the surface. The areas, normalized by the total coating area, are 36.3% for [Ag(5%)] and 36.9% for [Ag(22%)]. Consequently, Ag segregates the surface to form nanoparticles. Similar results were obtained for all the other coatings (data not shown).

### 2.2. Asssessment of Antibacterial Activity

For the antibacterial assays, the control samples are stainless steel coupons [SS], the carbon matrix without Ag doping a-C:H films [C] and a pure silver layer [Ag(100%)].

#### 2.2.1. Fluorescent Test and Counting CFU

**Figure 4:**
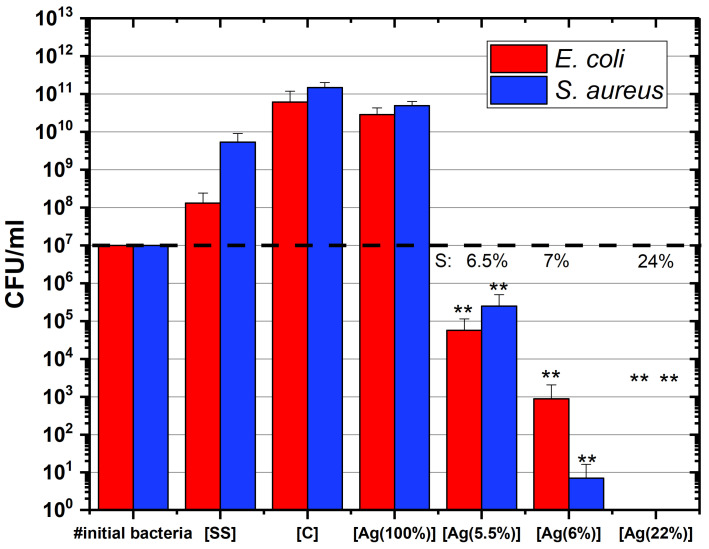
a-C:H:Ag coatings performance evaluated by a CFU method against *E. coli* (in red) and *S. aureus* (in blue) bacterial species during an incubation time of 24 h. Data are presented as the mean ± SD (n=3). Results of the Mann–Whitney statistical test are shown in the graph (** indicates *p* < 0.01). [SS] = stainless steel coupons (CTRL); [C] = hydrogenated amorphous carbon coating (CTRL); [Ag(100%)] = coating containing 100% of Ag (CTRL); [Ag(5.5%)] = the carbon matrix containing 5.5% of Ag in the bulk and 6.5% of Ag at the surface; [Ag(6%)] = the carbon matrix containing 6% of Ag in the bulk and 7% of Ag at the surface; [Ag(22%)] = the carbon matrix containing 22% of Ag in the bulk and 24% of Ag at the surface.

The cell viability was investigated by using a colony-forming unit (CFU) method. Figure 4 shows the number of CFU depending on the silver concentration in the coatings. The red and blue bars represent the results obtained on *E. coli* and *S. aureus*, respectively. The Ag surface concentrations of the samples are displayed above these bars (indicated by “S” on the graph). The samples which have a higher CFU count than the initial number of bacteria (indicated by a dashed line) are considered as non-antibacterial coatings, indicating a bacterial proliferation on the sample surface. This is the case for the control samples: [SS], [C] and [Ag(100%)]. In contrast, a lesser CFU count suggests that the considered sample shows antibacterial properties. The results obtained for the a-C:H:Ag coatings demonstrate that they have a bactericidal activity which depends on the silver concentration in the layer. At least a 2-log reduction in the number of bacteria, *E. coli* or *S. aureus*, can be observed between the initial number of bacteria in contact with the coated surfaces and the CFU count. As can be seen in this graph, the more the silver concentration, the more this log reduction is observed, suggesting a more pronounced antibacterial activity. For [Ag(22%)], no colony was counted after seeding on the LB agar plates, indicating that the antibacterial activity is substantial. As shown by Mann–Whitney statistical analysis, the reduction in the number of CFU is significant (p<0.01). It should also be mentioned that *S. aureus* are more resistant to silver than *E. coli* in the majority of the samples.

**Figure 5:**
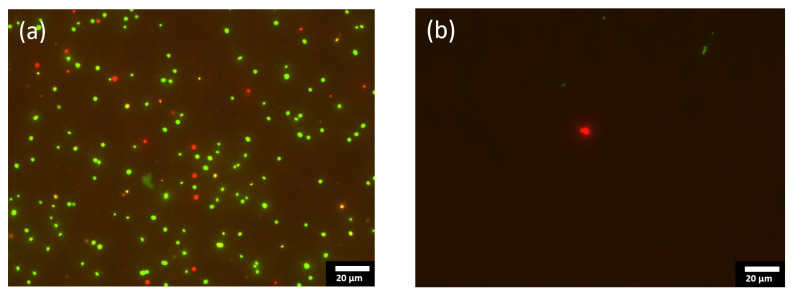
Fluorescent optical micrographs of the stainless steel substrate [SS] (**a**), and [Ag(22%)] (**b**), with bacterial species, *S. aureus*. These 2 pictures illustrate the results obtained by using the Live/Dead BacLight Bacterial Viability Kit. [SS] = stainless steel coupons (CTRL); [Ag(22%)] = the carbon matrix containing 22% of Ag in the bulk and 24% of Ag at the surface.

Figure 5 shows the fluorescent optical micrographs as obtained by using LIVE/DEAD bacterial viability kits on the [Ag(22%)] sample. That two-color fluorescence assay enables a direct counting of the bacterial viability based on the integrity of the cell membrane. The bacteria with an intact membrane are stained in green whereas the bacteria with an altered membrane appear in red. As can be seen, the number of green and red dots on the coated sample (Figure 5b) is far less than on the stainless-steel substrate (Figure 5a), which is known to not kill bacteria. Similar results were obtained with *E. coli* bacteria. The decrease in the number of altered red-stain bacteria and the decrease in the total number of stained bacteria (live and dead) not only demonstrates that a-C:H:Ag coatings kills Gram-negative and positive bacteria, but also hinders their growth.

#### 2.2.2. Multiple Exposures

**Figure 6:**
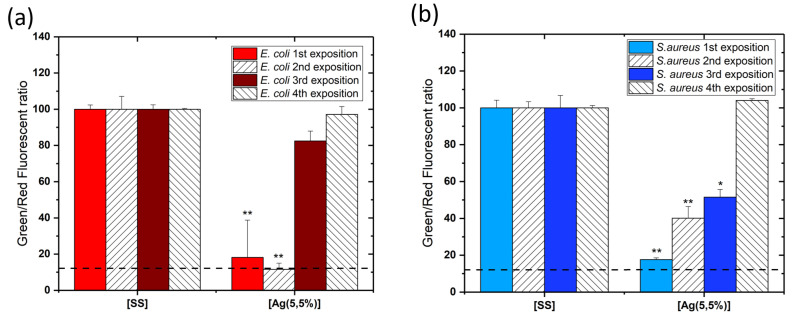
Diagram showing the standardized experimental results of the viability test using LIVE/DEAD BacLight Bacterial Viability Kits on the reference sample stainless steel substrate [SS] and the carbon matrix containing 5.5% of Ag [Ag(5.5%)]. Every exposure entails placement of a new set of fresh bacteria on the surface of the same coating. (**a**) shows results of *E. coli* (in red) and (**b**) *S. aureus* (in blue). Data are presented as the mean ± SD (n=3). Results of the Mann–Whitney statistical test are shown in the graph (* indicates *p* < 0.05 and ** indicates *p* < 0.01).

In order to evaluate the antibacterial efficiency of coatings after multiple exposures, the coated samples were exposed to bacteria 4 times. After each exposure, the samples were washed with water and sterilized prior to being exposed to another fresh set of bacteria. A cell viability assay was performed after each bacteria exposure. Figure 6 illustrates the results expressed as the ratio of the number of live cells to the number of dead cells for each exposure. Figure 6a shows the toxicity against the *E. coli* species (in red) and Figure 6b, against the *S. aureus* species (in blue). Data indicates that after 2 expositions, a significant antibacterial activity is still observed. After additional exposures, the viability of the bacteria increases, indicating that the antimicrobial effect is lost after multiple exposures. Indeed, after the 3rd exposure, the cell viability reached approximately 50% for *S. aureus* and more than 80% for *E. coli*, while the antibacterial activity of [Ag(5.5%)] completely disappears after the following exposures. It was also observed that the *S. aureus* species is more resistant than *E. coli*, with a-C:H:Ag films showing less activity against this latter species.

**Figure 7:**
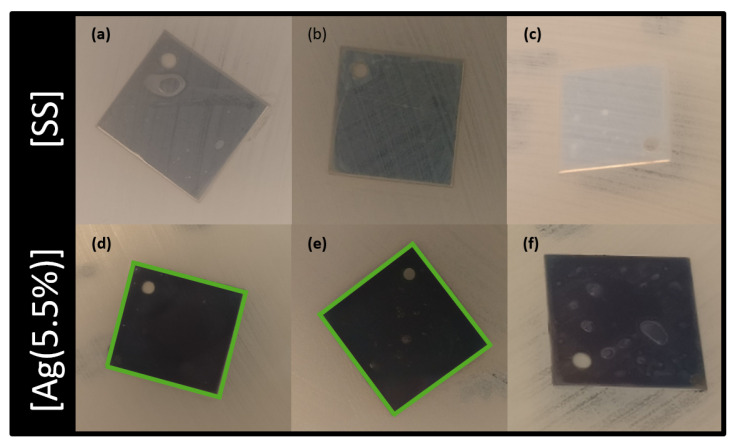
Optical micrographs of the modified antibiotic tests after multiple exposures: first column = first exposure; second column = second exposure, and third column = third exposure. Images (**a**–**c**) are associated with [SS] and (**d**–**f**) with [Ag(5.5%)]. The square size is 20 × 20 mm. [SS] = stainless steel coupons (CTRL); [Ag(5.5%)] = the carbon matrix containing 5.5% of Ag in the bulk and 6.5% of Ag at the surface.

In addition, Figure 7 exhibits the optical micrographs of a modified Kirby–Bauer test, which evaluates the diffusion of silver in a solid bacteria setting. The antibacterial activity is shown by the appearance of an inhibition zone in the vicinity of the coated sample: direct interaction with the coating indicates that Ag may be released and may diffuse in the solution. Stainless steel has no bactericidal features (Figure 7a–c) since no inhibition zones were detected. For the [Ag(5.5%)] samples (Figure 7d–f), the halo is located above the surface but also as a halo around the samples, indicating that silver is released and diffuses in the solid bacterial solution (represented in green). This diffusion is only located in the sample boundaries. After the 3rd exposure (Figure 7f), Ag is not released in the LB agar plaque. It also has to be noted that the coatings lose their antimicrobial properties with the number of exposures.

**Figure 8:**
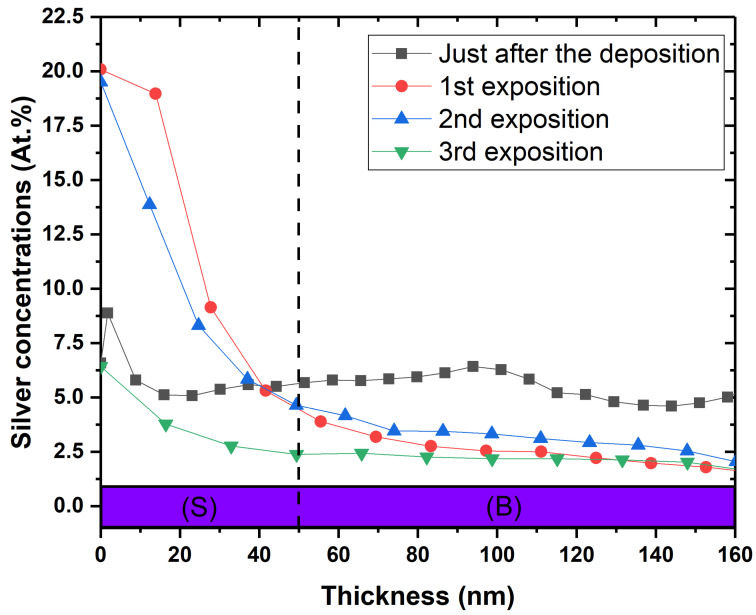
Silver depth profile of the [Ag(5.5%)] antimicrobial layer: before modified Kirby–Bauer assay (in grey), after the first bacterial exposure (in red), after the second bacterial exposure (in blue) and finally, the third bacterial exposure. These XPS profiles were measured on the samples used for the modified K-B test. (S) = surface ranging from 0 to 50 nm; (B) = bulk.

In order to confirm the hypothesis of silver release, the depth profile of silver concentration in the coatings was measured after multiple exposures. The results are shown in Figure 8 for the [Ag(5.5%)] sample. The concentration decreases from 6% to 2% in volume after four exposures, whereas at the surface, it increases after the first exposure and decreases thereafter. In addition, the characteristic silver peak of interface disappears. Silver seems to strongly segregate from the carbon matrix and diffuse towards the surface to interact with the bacteria. When the Ag reservoir is empty, the antibacterial capacity is reduced.

## 3. Discussion

This work aimed at assessing the antibacterial properties of silver-doped hydrogenated amorphous carbon coatings over multiple exposures to bacteria.

The CFU results prove that these coatings present an antibacterial effect, which non-linearly depends on the concentration of Ag in the antimicrobial layer, in accordance with results from the literature. Table 2 evidences that the antibacterial features of these a-C coatings doped with Ag depend on the Ag concentration in the antimicrobial layer. Dominguez-Meister et al. [27] suggested that there should be an Ag threshold above which antibacterial properties can be observed. They estimated it at 7 at.%. However, several groups obtained a near complete eradication of bacterial species with a lower concentration (5.5% in the present study; 6.5% in [28]). This threshold difference can be explained by the structure of the carbon matrix and hybridization level (ratio of sp^3^ and sp^2^ bonds) as well as by surface morphology such as the roughness, the number and the size of AgNPs [29]. However, the Ag concentration at the surface must also be taken into consideration. Cloutier et al. clearly showed the silver dependence in the antibacterial results: the more the at.% Ag decreases, the lower is the pronounced effect [30]. Although the films deposited by Kwok et al. contained a very large amount of Ag at the surface, they did not exhibit a high activity against the pathogens [31].

The main hypothesis related to the mechanism responsible for this antibacterial effect of a-C:H:Ag is silver mobility [32]. Interestingly, results in this study demonstrate movements of Ag in the support matrix in the absence of bacteria. Indeed, we showed a decrease in the silver content in the antimicrobial layer from 22% to 12% after 1 year (Figure 3). Since Ag is not miscible with carbon [33], the silver which is homogeneously deposited along the coating depth moves and accumulates at the surface, even at atmospheric temperature and humidity. This phenomenon is called surface segregation of silver from the carbon matrix. This leads to a high local silver concentration, which results in the formation of Ag nanoparticles driven by a self-assembling phenomenon. As time goes on, silver continues to accumulate at the surface, enabling nanoparticle growth and coalescence according to an Ostwald ripening phenomenon, as already described by Lucas et al. [34]. Many works have also revealed similar mobility of silver in a carbon matrix. Choi et al. showed that the silver atoms segregate as a second phase in an amorphous carbon matrix, forming clusters at the film surface [35]. Wang et al. [36] studied the local enrichment of Ag over time at the surface in films containing different concentrations. Their results evidenced that segregation is proportional to the silver content in the bulk. Moreover, this segregation can be influenced by the structure and the morphology of the carbon support matrix. Six months after deposition, no segregation was reported in a compact featureless morphology coating, while a columnar structure coating showed a uniform decrease in Ag content from 18 to 14 at.% in bulk [37]. The authors highlight that this reduction is correlated to an increase in surface coverage from 26% to 55%. All the aforementioned studies suggest that the driving force for Ag segregation is due to the structure and the morphology of the support matrix and a reduction in residual stresses.

**Figure 9:**
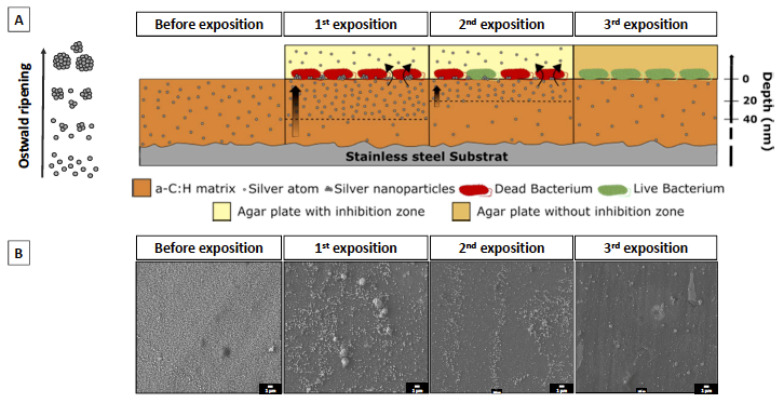
(**A**) Schematic description of the silver release mechanism and the effect on bacteria for multiple exposures. The silver homogeneously deposited along the coating depth moves and accumulates at the surface, leading to a high local silver concentration that results in the formation of Ag nanoparticles driven by a self-assembling phenomenon (Ostwald ripening phenomenon). (**B**) Representative SEM images of the coating surface before and after each bacterial exposure.

Moreover, a significant decrease in the Ag content was evidenced after incubation of coatings with bacteria, suggesting that the Ag is “pumped” away from the surface. When depleted, an Ag concentration gradient appears near the surface, promoting the Ag diffusion from bulk to surface, with, as a consequence, a refilling of the surface with Ag at the expense of the Ag concentration, which continuously decreases in the bulk. This takes place as long as the bulk of the coating can act as an Ag reservoir (Figure 9). This Ag release hypothesis corroborates the work of Dominguez-Meister, who quantified by ICP the time dependence of the Ag released from a-C films doped with Ag in deionized water [27]. The coating doped with 9.7% of Ag presents a high release during the first days to reach a maximum of 0.24 mg/L afterwards (estimated at 9% of the Ag total content contained in the antimicrobial layer), followed by a decrease in the delivery rate over the following days. However, as shown by Cloutier et al. the kinetics of release can be slower, suggesting a longer segregation effect. No peak and no concentration plateau were observed after an immersion of 2 weeks (cumulative Ag release = 1.5 µg/L) for a DLC coating doped with 1.7% Ag. Manninen et al. performed similar experiments with coatings immersed in 0.9% NaCl physiological solutions [38]. For a sample doped with 20 at.% Ag, the cumulative Ag release is linear and the maximum release is reached for 6 µg/cm^2^ (estimated at 1.75% of the total Ag content) after an immersion period of 168 h. There then ensues a slight decrease to 5 µg/cm^2^ during a month, suggesting a light stability in the Ag release. The assumptions made by Manninen et al. are that NaCl crystals form by precipitation, which reduces the Ag release.

The relation between this Ag mobility in the carbon matrix and the antibacterial effect of a-C:H:Ag coatings was studied by Cloutier, indicating a release of silver outside the coatings, demonstrated by a modified Kirby–Bauer assay. This effect on the solid bacterial solution seems localized at the surface of the film, showing a limited diffusion of silver for the coating containing from 0 to 2.4% of Ag in the antimicrobial layer. For the highest Ag concentration, Wang demonstrated that the coating containing more 6.1% presented a diffusion in the coating vicinity, indicating a greater diffusion after the same incubation time of 24 h at 37 °C [36]. The Ag release from our coatings corroborates these works with a localized effect at the surface with 5 at.% Ag. As illustrated by the depth profiles obtained by XPS after multiple bacterial exposures (Figure 8), the Ag is segregated towards the surface, released outside the coating and the volume of the coating acts as an Ag reservoir. The challenge for biocide-releasing coatings is to control the release kinetics of silver in time to obtain a long-term antibacterial effect.

Overall, our work highlights that the efficiency of antibacterial properties is affected by the AgNP content in the top surface of the antimicrobial layer, while the stability of this antibacterial effect with time is dependent on the Ag migration from the bulk (silver reservoir) to the coating surface. Therefore, good antibacterial carbon-based coatings should contain hydrogen and enable silver segregation toward the surface, and will be active as long as the bulk is not silver-depleted. Thick coatings with a few % of silver in the bulk and with an optimized microstructure to control silver diffusion toward the surface are the key parameters to produce active films against bacteria. This is summarized in the following figure.

## 4. Materials and Methods

### 4.1. Preparation and Production of Coated a-C:H:Ag Samples

The a-C:H:Ag were obtained by a low pressure discharge of C_2_H_2_ in a magnetron-based system equipped with Ag targets. The reader can refer to [39] for additional information about the PVD production process. During the discharge, the Ar/C_2_H_2_ ratio C_2_H_2_ was adjusted to enable Ag targets to be covered with a carbonaceous deposit. By adjusting the flow and power parameters, it was possible to adapt the Ag content in the carbon layer. During all the experiments, the substrates were biased at −175 V.

400 nm thick films were deposited on 20 × 20 mm chemically etched 304 stainless steel plates ($R_{a}<\;0.3\;\mu m$). The sample size was optimized to place a 50 µL droplet of bacterial solution on the coated surface. Thanks to the volume of the vacuum chamber, each production batch enabled the coating of more than 50 samples per deposition.

Prior to any deposition, the samples were cleaned in a heated ultrasonic bath in a Galvex and Rodaweg solution during 5 min and at 50 °C, and also etched in situ by an Ar glow discharge for 1800 s to remove native oxide layers.

### 4.2. Characterization of Samples

The surface morphology of the samples was observed by scanning electron microscopy (SEM—JEOL 7500-F), operating with 5–15 kV accelerating voltage for top and cross-section respectively.

The depth chemical compositions of the samples were measured by X-ray photoelectron spectroscopy (XPS—K-alpha spectrometer, Thermo Scientific, Waltham, MA, USA). The monochromatic X-ray source is the K-α line of aluminum (hv=1486.6 eV) at 1.5 k eV photon energy with a spot size of 250 × 250 µm^2^. The energy of the Ar^+^ sputtering beam for depth profiling is 2 k eV. The etching time was converted to depth assuming the sputtering rate of pure C.

The data were processed using the Avantage© software (Thermo Scientific, Waltham, MA, USA). Note that the H content cannot be measured using this technique, but was estimated in a prior work [26].

### 4.3. Assessment of Antibacterial Properties

#### 4.3.1. Growth of Bacterial Strains

Bacteria stock cultures of *Escherichia coli* (ATCC 25922) and *Staphylococcus aureus* (ATCC 25923) were obtained from −80 °C freezer stocks vials containing 25 beads in media rich in glycerol. After thawing and under aseptic conditions, a bead was removed with a sterile loop or needle and inoculated onto a Tryptic Soya Agar (TSA) plate. The plates were incubated for 24 h at 37 °C, with 5% CO_2_. After incubation, a single colony of each strain was inoculated in a LB broth and incubated 24 h at 37 °C, with 5% CO_2_.

Three different antibacterial assays were performed on the samples: two quantitative (CFU and LIVE/DEAD) and one qualitative (Kirby–Bauer).

#### 4.3.2. CFU and LIVE/DEAD

Under an air-flow hood, each sample was placed in a 6-well plate and sterilized with a 70% *v*/*v* ethanol solution during 2 h till total evaporation. The empty spaces between the wells were filled with sterile phosphate buffer saline (PBS) (Lonza Bioscience, Verviers, Belgium) to avoid media evaporation from the bacterial solution.

A droplet of fifty microliters of bacterial solution containing 10^7^ bacteria/mL of *E. coli* or *S. aureus* was placed on the surface of each sample and incubated at 37 °C overnight.

The droplet was collected with 450 µL of 0.85% NaCl solution:

- 100 µL were stained with a LIVE/DEAD BactLight Bacterial Viability kit (L7012 from Molecular probes) according to the manufacturer protocol and placed in a 96-well plate. The 96-well plate was read in fluorescence with a SpectraMax iD3 (Molecular Devices, San Jose, CA, USA). The excitation wavelength was 485 nm for both dyes. The emission wavelengths were 530 nm (SYTO9: live bacteria) and 630 nm (Propidium iodide: dead bacteria). The data acquisition was performed by using the SoftMax pro 7.1 software. To increase the statistical significance, each experiment was repeated three times in duplicate and each sample was analyzed twice.
- 100 µL of each sample from the 6-well plates described above (considered as the dilution 10^−1^) was collected and diluted in LB media from 10^−1^ to 10^−8^. 20 µL of each dilution was inoculated three times on LB agar square plates and incubated overnight at 37 °C, with 5% CO_2_. The plates were photographed and counted to determine the Colony Forming Units. To increase the statistical significance, each experiment was repeated three times in duplicate and each sample was inoculated three consecutive times for multiple exposure studies.

The antibacterial activity of the samples was calculated using the following Equation:

$$R\;\left(\%\right)=\;\frac{\left(N_{2}-N_{1}\right)}{N_{2}}\times 100,$$

where R is the antibacterial power in percentage (%); N_1_ and N_2_ are the mean numbers for the CFU count for samples containing silver and the control sample, respectively.

#### 4.3.3. Modified KIRBY–BAUER TEST (HALO TEST)

Under an air-flow cabinet, bacterial solutions of *E. coli* or *S. aureus* at 0.5 MacFarland were spread onto LB agar plates by using a sterile swab. The sterile swab was placed into the bacterial solution and gently pressed and rotated against the inside of the tube to remove the excess liquid. To obtain a uniform growth, the swab was streaked in one direction onto the whole plate, rotated at 90° and streaked again in one direction. This rotation was done 3 times. The plates were set to dry under the air-flow cabinet for 5–10 min. The samples were placed face down onto the inoculated plates, gently pressed and incubated overnight at 37 °C 5% CO_2_. After the incubation, the halo around the samples was observed and measured.

#### 4.3.4. Statistical Analysis

The statistical analysis of the obtained results was performed using a Mann–Whitney test (GraphPad Prism) with the [SS] control sample, namely, 304 stainless steel substrates, vs. each sample containing silver. *p* < 0.05 was chosen as the threshold of significance and significant differences of *p* < 0.01 and *p* < 0.05 were denoted with ** and *, respectively.

## 5. Conclusions

This work provides a better understanding of the behavior of a-C:H:Ag layers when exposed to Gram-positive or -negative bacteria. It highlights the need to develop thick coatings with silver in the bulk, acting as a reservoir, and to master the coating microstructure to control silver diffusion towards the top surface. These are the two key parameters for development of release Ag coatings to be applied on hospital fittings surrounding the patients in order to obtain a long-term antibacterial effect.

## Figures and Tables

**Table 1:** Particle-size distribution characteristics of the as deposited films.

Characteristics	[Ag(6%)]	[Ag(22%)]
Mean Feret diameter (nm)	59±20	53±40
Circularity	0.88	0.91
Particle density (Part./μm^2^)	49	188
Surface covered (%)	4.8	14.4

**Table 2:** Comparative table showing the experimental results of the antibacterial activity obtained by a CFU method for Ag-release coatings against *E. coli* strains. The antibacterial activity is expressed in % as seen in the section “Materials and methods”. A value of 100% means that the antibacterial activity is total: not one colony is counted after seeding. a-C = amorphous carbon; ta-C = tetragonal amorphous carbon; a-C:H = hydrogenated amorphous carbon. NP = nanoparticles.

Support Matrix	Ag Concentration (at.%) (S: Surface, B: Bulk)	Ag Morphology at Surface before Assay	Antibacterial Power (%)	Ref.
a-C	7.4 ^B^	NP	67.44	[27]
	12.6 ^B^	NP	96.94	
	23.4 ^B^	NP	85	
ta-C	70 ^S^	Not specified	98.12	
	70 ^S^	Not specified	99.98	[31]
	90 ^S^	Not specified	99.99	
a-C:H	6.5 ^B^	NP	93±2	[28]
	16.7 ^B^	NP	96±2	
	20 ^B^	NP	98±1	
	51.2 ^B^	NP	96±3	
	71.4 ^B^	NP	96±2	
a-C:H	0.2 ^S^	NP	60.0	[30]
	0.4 ^S^	NP	99.0	
	1.7 ^S^	NP	99.2	
	2.4 ^S^	NP	99.6	
a-C:H	6.5 ^S^–5.5 ^B^	NP	99.99	
	7 ^S^–6 ^B^	NP	99.99	This work
	24 ^S^–22 ^S^	NP	99.99	

^S^: Surface, ^B^: Bulk.

## Data Availability

Not applicable.
